# Differential effects of female aging on sympathetic blood pressure regulation at rest and during stress in humans

**DOI:** 10.14814/phy2.70347

**Published:** 2025-04-26

**Authors:** Christin Domeier, Thomas G. Bissen, Joseph D. Vondrasek, Matthew C. Babcock, Austin T. Robinson, Joseph C. Watso

**Affiliations:** ^1^ Cardiovascular and Applied Physiology Laboratory Florida State University Tallahassee Florida USA; ^2^ Department of Medicine University of Colorado – Anschutz Medical Campus Aurora Colorado USA; ^3^ Neurovascular Physiology Laboratory Indiana University Bloomington Indiana USA

**Keywords:** aging, apnea, cardiovascular, cold pressor test, sympathetic transduction

## Abstract

Older female (OF) adults exhibit blunted resting sympathetic blood pressure (BP) transduction compared with young female (YF) adults, affecting BP regulation. However, studies often lack control over health factors like body composition or habitual physical activity. Therefore, we compared resting sympathetic BP transduction and neurovascular responses during cold pressor test (CPT) and end‐expiratory apnea between YF (*n* = 12) and OF (*n* = 9) matched for several health factors. We measured beat‐to‐beat hemodynamics and muscle sympathetic nerve activity (MSNA). OF exhibited higher resting supine BP and MSNA (*p*_s_ < 0.001) than YF. OF exhibited blunted increases in mean BP and diastolic BP following spontaneous MSNA bursts at rest. During the CPT, OF exhibited a smaller percent increase in total MSNA (interaction effect: *p* = 0.001) but not MSNA burst frequency responses. Mean BP increases were not different between groups, but OF exhibited a higher ∆mean BP/∆MSNA burst frequency ratio (*p* = 0.003). During apnea, OF experienced a smaller percent MSNA increase in total MSNA (*p* < 0.05), larger mean BP increases (interaction effect: *p* = 0.031), and higher ∆mean BP/∆total MSNA ratio (*p* = 0.003). These findings suggest attenuated signal‐averaged sympathetic BP transduction at rest but increased time‐averaged sympathetic transduction to mean BP during cold and apneic laboratory stressors in OF.

## Graphical abstract

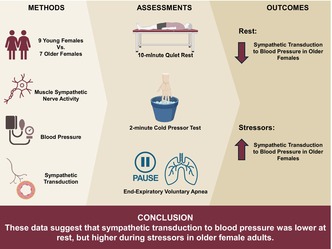

## 1 INTRODUCTION

Older female (OF) adults have the highest prevalence of hypertension among any age or sex group in the United States. This is alarming because high blood pressure (BP) is the leading modifiable risk factor for cardiovascular disease (CVD) (Martin et al., 2024). In addition, current projections indicate that the number of older adults worldwide will almost double by 2050. Therefore, the amount of CVD‐related morbidity and mortality is likely to increase dramatically (Heidenreich et al., 2011; Organization WH, 2015). Compared with younger females (YF), OF exhibit higher resting muscle sympathetic nerve activity (MSNA) and blunted sympathetic transduction of BP, suggesting reduced responsiveness of the vasculature to MSNA (D'Souza et al., 2023; Vianna et al., 2012). These prior studies assessed transduction by analyzing the increases in BP in response to spontaneously occurring MSNA bursts using signal‐averaging methods (Young et al., 2021). While the mechanism remains incompletely understood, the reduced vascular responsiveness to MSNA on a beat‐to‐beat basis may contribute to increased MSNA (Charkoudian et al., 2006; Hart et al., 2009). For example, aging attenuates vascular sensitivity to sympathetic stimulation when examined using local intra‐arterial infusions of the α‐adrenergic agonist phenylephrine (Kruse et al., 2018), which may be one reason MSNA increases with aging. Further, prior studies have demonstrated that reduced transduction is associated with higher MSNA, even in younger adults (Charkoudian et al., 2006; Nardone et al., 2021; Robinson et al., 2019).

In addition to signal‐averaging techniques to assess sympathetic transduction of BP, pressor responses to MSNA can be assessed during a stressor using time‐averaging methods (Ray & Monahan, 2002). For example, MSNA and BP increase during physiological stressors, such as the cold pressor test (CPT) (Victor et al., 1987) and end‐expiratory apnea (EEA) (Steinback et al., 2010). Including physiological stressors such as the CPT and EEA can aid in identifying augmented cardiovascular risk and changes in autonomic function with aging, providing a better understanding of how physiological stress impacts health (Barnett et al., 1963; Wood et al., 1984). Previous studies using the CPT reported no differences in BP responses between OF and YF participants, with mixed findings for MSNA responses (Keller‐Ross et al., 2020; Miller et al., 2019). It is worth noting the law of initial values may have influenced these findings (Berntson et al., 1994), such that the lack of difference in BP and MSNA responses in these studies may have been influenced by OF exhibiting directionally higher resting BP and MSNA (Keller‐Ross et al., 2020; Miller et al., 2019). Additionally, aging is associated with attenuated sympathetic recruitment during voluntary EEA (Badrov et al., 2016). However, these data were collected in a mixed population of male and female participants. The mixed findings and lack of information on the interplay between resting and CPT‐ or EEA‐evoked sympathetic transduction of BP highlight the need for further research on how female aging affects autonomic cardiovascular responses during physiological stress.

In addition to age, other factors, including body composition (Holwerda et al., 2023), hydration status (Watso & Farquhar, 2019), dietary sodium (Brooks & Osborn, 2012; de Boer et al., 2018), and habitual physical activity (Ehlers et al., 2023; Mortensen et al., 2014; Seals et al., 2008) affect sympathetic transduction of BP, BP reactivity, and/or MSNA reactivity. However, these factors were not always considered in previous studies comparing sympathetic transduction or BP and MSNA between YF and OF participants. Therefore, we sought to study YF and OF participants matched for body mass index (BMI), body fat percentage, hydration status, pre‐trial sodium intake, and habitual physical activity. We compared sympathetic transduction to BP at rest using signal‐averaging and during the CPT and EEA using time‐averaged BP and MSNA. We hypothesized that OF participants would exhibit (1) attenuated signal‐averaged sympathetic transduction of BP at rest and (2) attenuated MSNA and BP responses during the CPT and EEA as well as a blunted time‐averaged sympathetic transduction of BP, in part, due to higher resting MSNA and BP.

## 2 METHODS

### 2.1 Participants

Ethical approval for this study was obtained from the Institutional Review Board at the University of Delaware and the study was conducted following the Declaration of Helsinki. The data reported here were used for secondary analysis and are part of a larger registered trial (ClinicalTrials.gov Identifier: NCT03560869). All participants provided written consent before enrollment into the study. We measured seated BP in triplicate after at least 5 min of quiet rest and only individuals with systolic BP between 90 and 140 mmHg and diastolic BP between 50 and 90 mmHg were included. Additional inclusion criteria were age between 20 and 35 (YF) or age between 55 and 75 (OF) years, BMI <30 kg/m^2^, and no known overt cardiovascular, neurological, renal, or pulmonary diseases. Exclusion criteria were nicotine use within the past 12 months, previous diagnosis of hypertension, and past or current use of antihypertensive medications. After selecting participants from the larger trial that were matched for BMI, body fat percentage, pre‐trial sodium intake, and physical activity habits, 21 participants (12 YF, 9 OF) were included in the analysis. All OF participants were post‐menopausal (self‐report; time since menopause not collected). All YF participants were assessed during the early follicular phase of their menstrual cycle (self‐report). Given the challenges in recruiting OF with the desired characteristics, we initially included a larger pool of YF to ensure adequate matching across key variables. YF participants were systematically excluded one by one based on BMI, body fat percentage, pre‐trial sodium intake, and physical activity habits. This approach allowed for greater precision in group matching. This subgroup analysis is unique relative to prior reports from the larger study focused on how mild dehydration affects resting sympathetic regulation of BP among young adults (Watso, Robinson, Babcock, Migdal, Wenner, et al., 2020), sympathetic reactivity among young adults (Watso et al., 2019), and the exercise pressor reflex among YF and OF (Watso, Robinson, Babcock, Migdal, Witman, et al., 2020).

### 2.2 Variables matched between groups

We measured body height and mass and calculated BMI during the screening visit. We estimated body fat percentage using bioelectrical impedance (Tanita Body Composition Analyzer, Model TBF‐300A; Arlington Heights, IL). To avoid any confounding effects of hydration status, we instructed participants to consume 23 mL of H_2_O/kg body mass/day for the 3 days preceding the trial and 250 mL of H_2_O before arrival at the experimental visit. We assessed the urine specific gravity from a 24‐h urine sample and a pre‐trial spot urine sample. We also instructed participants on how to estimate food portion sizes and complete a 3 day food and fluid diary to ensure they maintained the recommended daily sodium intake of 2300 mg/day (Standing Committee on the Scientific Evaluation of Dietary Reference, 2005). We objectively assessed habitual physical activity during seven consecutive days using tri‐axial accelerometers (ActiGraph wGT3X‐BT, Pensacola, FL, USA). We determined the average daily moderate‐to‐vigorous physical activity duration and step counts after matching wear and non‐wear times between physical activity logs from the participants and the ActiLife software (ActiLife v6.13.4) (Watso, Babcock, et al., 2021).

### 2.3 Experimental visit

We instrumented participants with equipment to assess MSNA, beat‐to‐beat BP, and heart rate (HR) (described below). All testing was completed in the supine position. Participants rested quietly for 10 minutes in a temperature‐controlled room (22–24°C) for baseline measures. Next, participants completed a CPT, during which the participant's dominant hand was placed in ice water (~4°C) for 2 min. After at least 10 min of recovery, participants performed EEA, where participants were instructed to fill their lungs, exhale completely, and hold their breath as long as possible. Before each stressor, an additional two‐minute baseline was performed as a reference point for corresponding reactivity analysis (e.g., stressor minus preceding rest period, Δ).

### 2.4 Autonomic and cardiovascular measures

Ambulatory BP from self‐reported waking hours was assessed in all participants preceding the experimental visit. The monitor measured BP on the non‐dominant arm every 20 min during the day. Data were included in the analysis if we obtained at least 15 measurements during wake time (Watso, Robinson, Babcock, Migdal, Wenner, et al., 2020).

As previously described (Robinson et al., 2019; Watso et al., 2019, 2022; Watso, Huang, et al., 2021), we measured beat‐to‐beat BP and Modelflow‐derived cardiac output (De Vaal et al., 2005; Sugawara et al., 2003) at the finger of the participant's non‐dominant hand using photoplethysmography (Finometer PRO; Finapres Medical Systems, the Netherlands) (Guelen et al., 2003). We followed the manufacturer's recommendation calibration procedures and used a height correction unit to account for the pressure difference due to height between the heart and the site of recording (finger) for each participant. Total vascular conductance was calculated by cardiac output/mean BP. We measured HR continuously using a single‐lead electrocardiogram (Dash 2000; GE Medical Systems, WI, USA).

We obtained integrated MSNA recordings from a tungsten microelectrode inserted in the peroneal nerve using standard microneurography technique (Hart et al., 2017; White et al., 2015). The criteria for an adequate MSNA signal included: (1) tapping of the muscles or tendons innervated by the nerve produced afferent mechanoreceptor discharges; (2) apnea produced an increase in MSNA; (3) stroking of the skin did not produce any afferent activity; and (4) sudden, unexpected stimuli (shout or clap) did not produce any increases in sympathetic activity. The nerve signal was amplified (80,000–90,000 times), bandpass filtered (700–2000 Hz), and integrated (0.1 s time constant) using a nerve traffic analyzer (model 662c‐4, Nerve Traffic Analyzer, University of Iowa Bioengineering, Iowa City, IA). The mean voltage neurogram was analyzed on a beat‐to‐beat basis to determine the presence or absence of MSNA bursts using LabView software and visual inspection. Based on recent guidelines (Hart et al., 2017; Shoemaker et al., 2018; White et al., 2015), the following criteria were used for identifying MSNA bursts: (1) >3:1 signal‐to‐noise ratio, (2) burst morphology consistent with MSNA bursts, and (3) a pulse‐synchronous signal. For indices of sympathetic outflow, MSNA was quantified as burst frequency (bursts/min), burst amplitude (a.u.), and total activity (burst frequency*burst amplitude) after normalization (assigning the largest MSNA burst value of 100 a.u.).

### 2.5 Statistical analysis

We used unpaired, two‐tailed t‐tests to compare group differences in baseline characteristics for normally distributed data (Shapiro–Wilk test *p* > 0.05) and two‐sided Mann–Whitney *U* tests for non‐normally distributed data (Shapiro–Wilk test *p* ≤ 0.05). To ensure group matching, we used equivalence testing to compare participant characteristics between age groups (Lincoln et al., 2022). For effect size estimation, we calculated Cohen's *d* for *t*‐tests and rank biserial correlation for Mann–Whitney *U* tests.

For resting sympathetic BP transduction BP, we analyzed the average change in BP for ten cardiac cycles after an MSNA burst (i.e., signal‐averaged sympathetic transduction) or non‐bursts using mixed‐effect models (group*time[repeated measure]). This approach aims to provide more insight into the association between sympathetic bursts and changes in BP under resting conditions. For mixed‐effect models, we calculated partial eta squared as a measure of effect size.

We also used mixed‐effect models to compare group differences in baseline and reactivity data between groups with mixed‐effect models. We calculated time‐averaged sympathetic transduction of BP during each stressor using the before‐mentioned time points as sympathetic‐pressure ratios (∆mean BP/∆MSNA burst frequency & ∆mean BP/∆MSNA total activity). This approach aims to assess the effects of sympathetic transduction across different stressors (CPT and EEA), and how this may differ with female aging. Significance was set a priori to *α* ≤ 0.05.

## 3 RESULTS

### 3.1 Participant characteristics

By design, BMI, body fat percentage, hydration status, physical activity, and pre‐trial sodium intake were not different and were equivalent between age groups (Table 1). Also, body mass, ambulatory awake systolic BP, and ambulatory awake diastolic BP were not different and were equivalent between groups (Table 1). All variables in Table 1 that were equivalent between groups for the whole cohort remained similar between age groups when examining the subset of individuals with MSNA data (∆_Lower_ *p* ≥ 0.15; ∆_Upper_ *p* ≥ 0.07).

**TABLE 1:** Participant characteristics.

	YF	OF	*p*‐Value (different)	Effect size	Equivalence testing		
					Δ_Lower_ *p*‐value	Δ_Upper_ *p*‐value	90% confidence interval
*Demographics*							
Sample size	12	9	‐				
Age (years)	22 [3]	64 [12]	**<0.001**	*r*_bc_ = 1.000	**<0.001**	1.000	−47.4 to −39.1
Body mass (kg)	63.4 ± 9.6	58.6 ± 8.1	0.236	*d* = 0.546	0.863	0.091	−1.8 to 11.5
Body mass index (kg/m^2^)	23.6 ± 3.6	22.8 ± 2.8	0.579	*d* = 0.255	0.360	0.369	−2.5 to 2.4
Body fat (%)	27.9 ± 6.0	31.3 ± 5.8	0.208	*d* = −0.578	0.077	0.859	−8.0 to 1.1
*Hydration, dietary sodium, and habitual physical activity*							
Spot urine specific gravity	1.015 ± 0.006	1.013 ± 0.005	0.311	*d* = 0.333	**<0.001**	**<0.001**	−0.003 to 0.007
24‐h urine specific gravity	1.013 ± 0.004	1.011 ± 0.002	0.432	*d* = 0.500	**<0.001**	**<0.001**	−0.001 to 0.004
Reported sodium intake (mg/day)	2275 [494]	2227 [542]	0.277	*r*_bc_ = 0.296	0.525	0.472	−284.0 to 307.0
24‐h sodium excretion (mg/day)	2499 [4399]	2437 [1254]	0.422	*r*_bc_ = 0.222	0.949	0.051	−10.0 to 2839.0
Moderate‐to‐vigorous physical activity (minutes/day)	80 ± 27	70 ± 35	0.547	*d* = 0.317	0.723	0.257	−17.4 to 37.2
*Cardiovascular and sympathetic measures*							
Ambulatory Systolic BP (mmHg)	118 ± 9	126 ± 14	0.138	*d* = −0.711	0.064	0.913	−18.0 to 1.3
Ambulatory Diastolic BP (mmHg)	69 ± 4	74 ± 11	0.173	*d* = −0.640	0.083	0.878	−12.4 to 1.72
Heart rate (bpm)	60 ± 7	62 ± 9	0.626	*d* = −0.215	0.273	0.630	−8.0 to 4.6
Finometer systolic BP (mmHg)	108 ± 7	145 ± 22	**<0.001**	*d* = −2.212	**<0.001**	0.999	−50.2 to −22.2
Finometer mean BP (mmHg)	77 [6]	101 [21]	**<0.001**	*r*_bc_ = 0.815	**<0.001**	0.999	−30.1 to −12.5
Finometer diastolic BP (mmHg)	63 ± 4	74 ± 10	**0.004**	*d* = −1.369	**0.006**	0.991	−16.9 to −4.1
MSNA (bursts/min)	13 ± 4	35 ± 6	**<0.001**	*d* = −4.229	**<0.001**	1.000	−25.9 to −14.5
MSNA (total activity; a.u.)	134 [140]	1096 [568]	**0.005**	*r*_bc_ = 0.810	**0.025**	0.975	−1773.0 to −180.0

*Note*: Characteristics of young female (YF) and older female (OF) participants. Values are shown as mean ± standard deviation (SD) or median [IQR; interquartile range]. Effect sizes are Cohen's *d* (*d*) or Rank‐Biserial Correlation Coefficient (*r*_bc_). Significance was set a priori to α ≤ 0.05.

Abbreviations: BP, blood pressure; MSNA, muscle sympathetic nerve activity.

### 3.2 Resting measures

**FIGURE 1:**
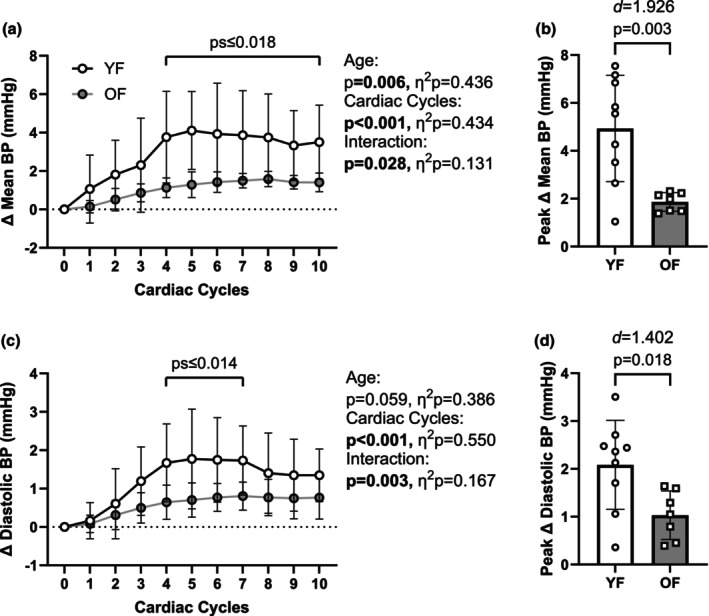
Resting Sympathetic Transduction to Blood Pressure (BP) following Spontaneous Sympathetic Bursts. (a) Mean BP increased following spontaneous bursts of muscle sympathetic nerve activity (MSNA) in both groups, with greater increases in young females (YF) during cardiac cycles 4–10. (b) Peak mean BP responses were higher in YF compared to OF. (c) Diastolic BP increased following spontaneous bursts of MSNA in both groups, with greater responses in YF during cardiac cycles 4–7. (d) Peak diastolic BP responses were higher in YF than in OF.

**FIGURE 2:**
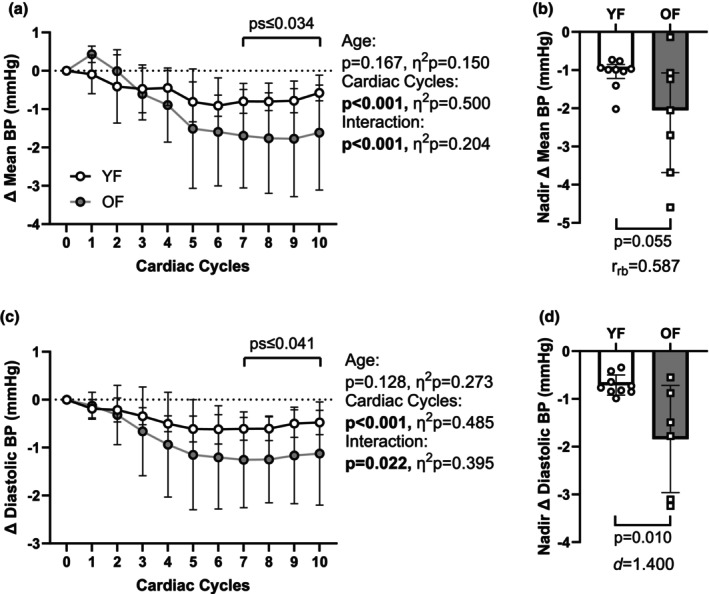
Resting Sympathetic Transduction to Blood Pressure (BP) following Non‐Burst Cardiac Cycles. (a) Reductions in mean BP during non‐burst cardiac cycles were greater in OF during cardiac cycles 7–10. (b) The nadir mean BP was lower in OF compared to YF. (c) Reductions in diastolic BP following non‐burst cardiac cycles were greater in OF during cardiac cycles 7–10. (d) The nadir diastolic BP was lower in OF than in YF.

We obtained resting finometer‐derived systolic BP, mean BP, and diastolic BP in all 21 participants, which were higher in OF compared to YF participants (Table 1). Heart rate was not different and was equivalent between groups (Table 1). We obtained adequate MSNA recordings in 16 (9 YF, 7 OF) of 21 participants at rest. MSNA burst frequency and MSNA total activity were higher in OF than in YF (Table 1). Mean BP increases following spontaneous bursts of MSNA were lower in OF during cardiac cycles 4–10 (Figure 1a). Additionally, the peak mean BP increases following spontaneous bursts of MSNA were lower in OF (Figure 1b). Diastolic BP increases following spontaneous bursts of MSNA were lower in OF during cardiac cycles 4–7 (Figure 1c). Additionally, the peak diastolic BP increases following spontaneous bursts of MSNA were higher in YF (Figure 1d). Reductions in mean (Figure 2a,b) and diastolic (Figure 2c,d) BP following non‐burst cardiac cycles were greater in OF during cardiac cycles 7–10.

### 3.3 Autonomic and cardiovascular reactivity during the CPT

**FIGURE 3:**
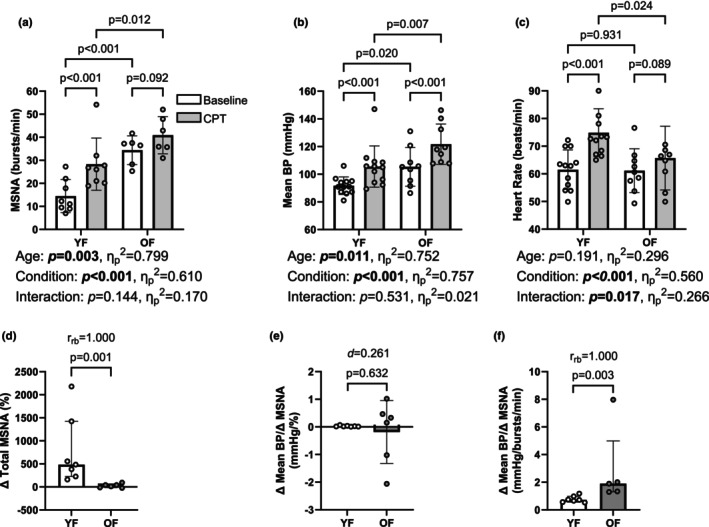
Autonomic Cardiovascular Reactivity to the Cold Pressor Test (CPT). Absolute MSNA burst frequency was higher in OF during baseline and CPT. There was no interaction effect; however, the partial eta‐square value of 0.170 indicates a medium effect size (a). The mean blood pressure (BP) during baseline and CPT was higher in OF, but there was no interaction effect of age and condition (baseline vs. CPT) (b). Heart rate (HR) increased more in YF (c). The relative muscle sympathetic nerve activity (MSNA) during CPT was greater in young females (YF) compared to older females (OF) (d). The ∆mean BP/∆relative MSNA ratio was not different between groups (e). The ∆mean BP/∆MSNA burst frequency ratio was higher in OF (f).

We maintained adequate MSNA recordings in 13 (7 YF, 6 OF) participants during the CPT. Total MSNA was higher in OF participants during baseline (YF: 116[142]; OF: 1149[1126] a.u.; *p* = 0.0012) and still tended to be higher during the CPT (YF: 629[812]; OF: 1660[1224] a.u.; *p* = 0.0734). However, the percent increase in total MSNA during the CPT was greater in YF (Figure 3d). Older female participants exhibited higher MSNA burst frequency during baseline and CPT compared to YF, but there was no interaction effect between age group and time point (Figure 3a). However, increases in burst amplitude were not different between groups (YF: ∆4 ± 16; OF: ∆4 ± 5 a.u.; *p* = 0.9814). Mean BP during baseline and the CPT was higher in OF participants and increased in both groups, but there was no interaction effect between age group and time point (Figure 3b). During the CPT, heart rate increased only in YF. Further, we found an interaction between age group and time point (Figure 3c). Similarly, cardiac output increased more in YF than in OF (YF: ∆1.0 ± 0.9; OF: ∆0.2 ± 0.5 L/min; *p* = 0.0191; *d* = 1.179), while the increase in total vascular conductance was different between groups (YF: ∆1.2[5.8]; OF: ∆‐4.9[3.7] ml/min/mmHg; *p* = 0.0184; r_bc_ = 0.61). The ∆mean BP/∆relative MSNA ratio was not different between groups during the CPT (Figure 3e). However, the ∆mean BP/∆MSNA burst frequency ratio was higher in OF (Figure 3f). We excluded one OF from this analysis because the 28 mmHg/burst/min value was more than 2.5 times the IQR above the median (Linder et al., 2023).

### 3.4 Autonomic and cardiovascular reactivity during EEA

**FIGURE 4:**
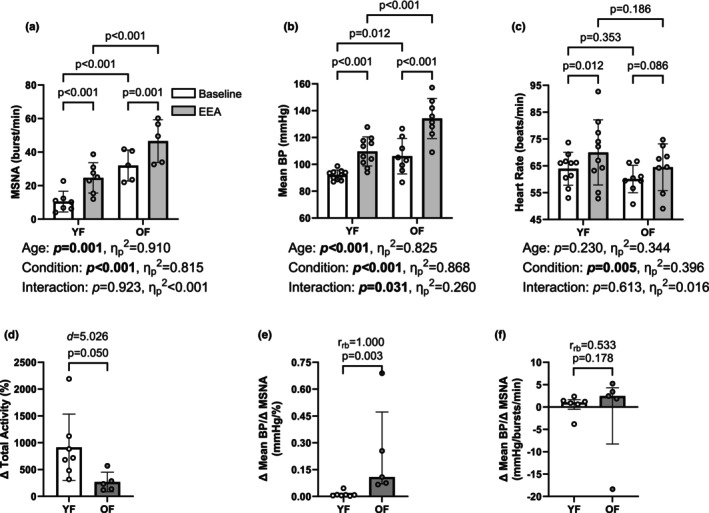
Autonomic Cardiovascular Reactivity to End‐Expiratory Apnea (EEA). Absolute MSNA burst frequency was higher in OF during baseline and CPT, but there was no interaction effect (a). The mean blood pressure (BP) during baseline and CPT was higher in OF, with an interaction between group (YF vs. OF) and condition (baseline vs. EEA) (b). Heart rate (HR) did not differ between groups (c). The relative muscle sympathetic nerve activity (MSNA) during CPT was greater in young females (YF) compared to older females (OF) (d). The ∆mean BP/∆relative MSNA ratio was higher in OF (e). The ∆mean BP/∆burst frequency ratio was not different between age groups (f).

Eighteen (10 YF, 8 OF) individuals completed EEA (*n* = 3 not completed due to time constraints); we maintained adequate MSNA recordings in 12 (7 YF, 5 OF) participants. The mean breath‐hold time was shorter in YF (YF: 26 ± 6; OF: 48 ± 16 s; *p* = 0.0013). Nonetheless, during the final 15 s of EEA, the percent change in total MSNA was lower in OF participants (Figure 4d), while increases in burst amplitude were not different between groups (YF: ∆30 ± 45; OF: ∆25 ± 22 a.u.; *p* = 0.8226). The absolute MSNA burst frequency was higher in OF participants during baseline and EEA and increased in both groups (Figure 4a). Similarly, mean BP was higher in OF during baseline and EEA. Further, mean BP increased to a greater extent in OF, based on an interaction between age group and condition (Figure 4b). Heart rate responses during the EEA did not differ between groups (Figure 4c). Interestingly, cardiac output increased in YF and decreased in OF during the last 15 s of EEA (YF: ∆0.3 ± 0.3; OF: ∆‐0.3 ± 0.6 L/min; *p* = 0.0157; *d* = 1.24), while total vascular conductance tended to decrease more in OF (YF: ∆‐5.7[8.2]; OF: ∆‐10.8[4.5] ml/min/mmHg; *p* = 0.0545; *r*_bc_ = 0.55). OF had a higher ∆mean BP/∆relative MSNA ratio than YF (Figure 4e). However, the ∆mean BP/∆burst frequency ratio was not different between age groups (Figure 4f).

## 4 DISCUSSION

### 4.1 Primary findings

In agreement with our first hypothesis, we found attenuated signal‐averaged sympathetic transduction of BP at rest in OF participants. In partial agreement with our second hypothesis, we observed (a) attenuated total MSNA, but not BP, responses during the CPT and (b) attenuated total MSNA and augmented mean BP responses during the EEA in OF participants. Our findings are strengthened by having participants across age groups well matched for body composition, hydration status, pre‐trial sodium intake, and habitual physical activity. These findings suggest a discordant effect of female aging on rest and reflex sympathetic transduction of BP. Interestingly, during the CPT, the ∆mean BP/∆burst frequency ratio was higher, but the ∆mean BP/∆relative MSNA ratio was not higher in OF participants. In contrast, the ∆mean BP/∆relative MSNA ratio was higher in OF participants during EEA but not the ∆mean BP/∆burst frequency ratio. Future work should examine sympathetic recruitment strategies (i.e., MSNA action potential counts, patterns, and synchronicity) to better understand the discordance of the interplay of MSNA and BP responses between stressors in female aging.

### 4.2 Resting sympathetic transduction to BP

We found that OF exhibited an attenuated increase in BP following spontaneous bursts of MSNA compared with YF. These data agree with three past reports (D'Souza et al., 2022, 2023; Vianna et al., 2012) suggesting that aging is associated with lower signal‐averaged sympathetic transduction of BP. Of note, only two of the three articles reported data among female‐only cohorts. Further, while each investigation controlled for some variables between age groups, no studies matched age groups for the variety of factors considered in the present analysis. Thus, our data advance our understanding of female aging and resting sympathetic regulation of BP by demonstrating attenuated sympathetic transduction of BP at rest in OF in the context of controlling for several health behaviors. The underlying mechanism for this phenomenon is not well understood. It has been hypothesized that age‐related reductions in postjunctional α‐adrenergic responsiveness lead to a reduction in the vasoconstrictive response after MSNA bursts rather than a decrease in norepinephrine release per MSNA burst (Charkoudian et al., 2006; Davy et al., 1998; Dinenno et al., 2002; Kruse et al., 2018). However, the mechanisms for reduced vasoconstrictive responsiveness in older females still need further assessment.

We found a greater decrease in mean and diastolic BP after cardiac cycles without bursts of MSNA in OF compared to YF, supporting D'Souza and colleagues' (2023) findings of a similar decrease in mean BP in OF. Thus, sympathetic vasoconstrictor activity appears important in OF to maintain beat‐to‐beat BP at rest. In conjunction with the observed attenuated rise in mean BP following MSNA bursts, these findings indicate a greater reliance on sympathetic activity for beat‐to‐beat BP regulation in OF. The higher sympathetic activity in OF at rest, reflected by a higher burst frequency, might counteract reduced vascular responsiveness to maintain homeostasis. Higher resting sympathetic activity is positively correlated with higher resting BP in OF but not in YF (Narkiewicz et al., 2005), which aligns with the higher resting MSNA and BP observed in OF in this study.

### 4.3 Autonomic and cardiovascular reactivity during the CPT

The CPT induces vasoconstriction because it is perceived as painful and stimulates cold thermoreceptors within the skin, which activate neural pathways and regions in the brain (e.g., rostral ventrolateral medulla) to increase MSNA (Hendriks‐Balk et al., 2020). CPT pressor responses predict future hypertension risk, with greater increases in BP during the CPT indicating a higher future risk of hypertension (Menkes et al., 1989; Wood et al., 1984). In this study, the relative change in total sympathetic activity was higher in YF compared to OF during the CPT. This heightened sympathetic response in YF was primarily driven by a relatively larger increase in burst frequency rather than burst amplitude. Although there was not an interaction between age group and condition for burst frequency, the large effect size suggests notable differences between age groups. Specifically, YF exhibited a higher sympathetic response, while BP reactivity was similar between both groups.

Despite higher sympathetic responses in YF during CPT, there were no differences in mean BP changes between groups. The exclusion of one outlier did not change that (*p* = 0.0774). However, the substantial effect size (*d* = 0.782) suggests that OF tended to have higher mean BP increases (YF: ∆11 vs. OF: ∆15 mmHg on average). This partially aligns with previous studies that did not exhibit a difference in mean BP changes during the CPT between groups (Keller‐Ross et al., 2020; Miller et al., 2019). However, this study found higher changes in relative MSNA in YF than in OF, potentially due to matching for variables that can influence sympathetic activity (e.g., BMI, sodium intake, etc.). Future studies in larger populations should continue to match for confounding factors to refine our understanding of autonomic BP regulation during stressors.

Sympathetic outflow increases BP mostly by decreasing total vascular conductance, but also by contributing to higher cardiac output during the CPT (Watso, Huang, et al., 2021). Interestingly, we found larger increases in cardiac output in YF, but larger decreases in total vascular conductance in OF. This suggests that the increase in mean BP was mainly driven by an increase in total peripheral resistance in OF and, to a greater extent, by an increase in cardiac output in YF. This aligns with the results of a meta‐analysis by Le et al. (2023), examining the contributions of cardiac output and systemic vascular resistance to increased blood pressure in children, and young and older adults. Their results suggest a greater contribution of cardiac output in young and vascular resistance in older adults (Li et al., 2023). The greater contribution of vascular resistance compared to cardiac output to increased blood pressure in older adults was additionally observed in other studies (Barnes et al., 2014; Tanaka et al., 2016). In addition, we extend these findings by looking at the association between sympathetic and cardiovascular responses (Figure 2). Sympathetic transduction to BP during the CPT, assessed by the ∆mean BP/∆relative MSNA ratio, was not different between groups. However, OF exhibited a higher ∆mean BP/∆burst frequency ratio. This reflects the smaller increases in burst frequency in OF during the CPT, but a lack of differences for increases in mean BP between groups.

### 4.4 Autonomic cardiovascular reactivity during EEA

EEA stimulates peripheral chemoreceptors in the carotid bodies and aortic arch by elevating the partial pressure of carbon dioxide (CO_2_), leading to increased MSNA and BP (Busch et al., 2019; Guyenet, 2014; Heusser et al., 2009; Leuenberger et al., 2001). In this study, YF exhibited a higher sympathetic response to EEA, reflected by higher relative increases in total MSNA, driven by the relatively higher increase in burst frequency rather than burst amplitude. On the contrary, OF exhibited greater BP reactivity, as demonstrated by the interaction between age group and time point. Our findings partially agree with previous studies that exhibited no difference in MSNA burst frequency changes during EEA between groups (Miller et al., 2019). However, this study found higher changes in relative MSNA in YF than in OF during EEA, potentially due to matching for variables such as BMI and habitual physical activity that influence sympathetic activity.

Breath‐hold time was longer in OF compared to YF. This might suggest a decline in chemosensitivity with age. However, by using the last 15 seconds of EEA, we aimed to account for potential age‐related differences in chemosensitivity. Similar to our finding during the CPT, we found larger increases in cardiac output in YF, but larger decreases in total vascular conductance in OF. This indicates that the increase in mean BP was mainly driven by the rise in total peripheral resistance in OF and, to a greater extent, by an increase in cardiac output in YF. In addition, we extend these findings by examining the association between sympathetic and cardiovascular responses (Figure 4). Contrary to our findings during the CPT regarding sympathetic transduction to BP, the ∆mean BP/∆burst frequency was not different between groups. However, OF exhibited a higher ∆mean BP/∆relative MSNA ratio. This reflects the smaller relative increases in total MSNA, but a greater increase in mean BP in OF.

The exact mechanism behind this pattern is unclear and cannot be fully explained by this study. However, previous research suggests that chronically elevated sympathetic activity and reduced β‐adrenergic mediated vasodilation in OF participants could increase sympathetic tone and arterial stiffening, contributing to elevated BP (Adams et al., 2023; Charkoudian et al., 2006; D'Souza et al., 2022; Hart et al., 2011). In addition, it has been suggested that the threshold of the baroreflex is shifted in older populations, and there might be alterations in the central processing that lead to exaggerated BP responses (D'Souza et al., 2022). Nevertheless, questions about the central and peripheral mechanisms of BP regulation in female aging remain unanswered and require further investigation.

### 4.5 Physiological relevance

Assessing sympathetic activity during rest and in response to physiological stimuli can inform us about cardiovascular function. The observed age‐related decline in sympathetic transduction at rest aligns with previous studies and may be attributed to a reduced responsiveness of α‐adrenergic receptors, potentially contributing to a compensatory increase in sympathetic outflow to maintain BP among OF. Interestingly, our analyses suggest a potential augmentation in sympathetic transduction during physiological stressors. This observation may be linked to reduced levels of vasodilatory mediators in postmenopausal female adults, such as estrogen and nitric oxide. Understanding the mechanisms underlying age‐related alterations in BP regulation among females is crucial for developing, implementing, and improving effective preventive care and treatment strategies to enhance cardiovascular health in aging females. Future investigation into these areas is warranted.

### 4.6 Limitations

1) Despite our exclusion criteria of seated resting systolic BP over 140 mmHg for both groups in a clinical exam room, which was assessed during an initial screening visit, resting supine BP was higher in OF during the experimental visit. This finding aligns with prior studies demonstrating BP can increase in the supine versus seated position (Eşer et al., 2007). We observed this particularly in OF, likely due to age‐related changes in vascular tone, baroreflex sensitivity, or other factors (e.g., cardiac filling) that need further assessment. However, it is important to note that the ambulatory awake BP data suggest that the older females did not have hypertension (defined as 24‐hour BP >135/80 mmHg). 2) Our sample size was modest (*n* = 12 YF & *n* = 9 OF), but similar to that of past reports examining resting and reflex sympathetic transduction of BP in female aging (median sample size among five most similar studies: *n* = 13 YF & *n* = 10 OF (D'Souza et al., 2022; D'Souza et al., 2023; Keller‐Ross et al., 2020; Miller et al., 2019; Vianna et al., 2012). For sympathetic transduction during EEA, the final stressor, the sample size decreased (*n* = 7 YF & *n* = 5 OF). However, to our knowledge, we provide the first age comparison for resting and reflex sympathetic transduction of BP with several important variables like body composition and relevant health behaviors, matched between age groups. 3) We observed greater baseline MSNA in OF, which may have influenced our results. A previous study reported reduced sympathetic, but not cardiovascular, reactivity during the CPT in OF with high baseline MSNA compared to low baseline MSNA (Akins et al., 2023). This suggested greater reflex sympathetic transduction in OF with higher baseline MSNA, which aligns with our findings. However, future research should assess sympathetic transduction and identify sympathetic recruitment strategies between OF and YF matched for baseline MSNA.

## 5 CONCLUSION

Among groups matched for BMI, body fat percentage, hydration status, pre‐trial sodium intake, and habitual physical activity, OF participants exhibited attenuated signal‐averaged sympathetic transduction to BP at rest compared to YF. During CPT and EEA, OF participants generally exhibited attenuated sympathetic responses but slightly greater BP responses compared to YF. Unexpectedly, we found increased ∆ time‐averaged sympathetic transduction to mean BP in OF participants during both stressors. Future work should examine sympathetic recruitment strategies (i.e., MSNA action potential counts, patterns, and synchronicity) to better understand the discordance between resting and reflex sympathetic regulation of BP in female aging in humans.

## AUTHOR CONTRIBUTIONS

MCB, ATR, JCW contributed to study design; all authors contribute to the acquisition, analysis, or interpretation of data; CD drafted the manuscript and all authors revised it critically for important intellectual content. All authors approved the final version of the manuscript. All authors agree to be accountable for all aspects of the work in ensuring that questions related to the accuracy or integrity of any part of the work are appropriately investigated and resolved. All persons designated as authors qualify for authorship, and all those who qualify for authorship are listed.

## FUNDING INFORMATION

CD is supported by the Florida State University Graduate School Legacy Fellowship. MCB is supported by the National Institutes of Health (NIH; K01HL164978). ATR is supported by the NIH (K01HL147998). JCW is supported by the NIH (K01HL160772) and the American Heart Association (23CDA1037938).

## Data Availability

Data are available upon reasonable request to the principal investigator after institutional data transfer agreement approvals.
